# Perspectives of Academic Oncologists About Offering Expanded Access to Investigational Drugs

**DOI:** 10.1001/jamanetworkopen.2022.39766

**Published:** 2022-11-01

**Authors:** Patrick Gould, Tasnim Salam, Laura Kimberly, Alison Bateman-House, Holly Fernandez Lynch

**Affiliations:** 1 Perelman School of Medicine, University of Pennsylvania, Philadelphia; 2 NYU Grossman School of Medicine, New York, New York

## Abstract

**Importance:**

The expanded access (EA) pathway permits patients to be treated with investigational medical products outside clinical trials. Because cancer care is a common indication for which EA is sought and these efforts require physician management, understanding oncologists' perspectives can help illuminate factors influencing patient access.

**Objective:**

To learn how oncologists practicing at academic medical centers (AMCs) perceive EA and their role in offering it.

**Design, Setting, and Participants:**

This qualitative study used data from semistructured interviews conducted from February 2020 to September 2021 with a purposive sample of oncologists recruited from large, urban AMCs in the northeast United States. Oncologists who had submitted at least 1 single-patient EA request to the institutional review boards at the University of Pennsylvania, Children’s Hospital of Philadelphia, NYU Langone Health, and Dana-Farber Cancer Institute from January 1, 2014, through January 31, 2020, were eligible to participate. Data were analyzed from July 2021 to March 2022.

**Main Outcomes and Measures:**

Interviews focused on oncologist practice demographics, experience with EA, factors relevant to decisions to pursue EA and comfort with those decisions, perspectives on oncologists’ role in EA, perspectives on the FDA’s role, and the Right to Try pathway to access investigational drugs.

**Results:**

Eligible oncologists were interviewed until thematic saturation was reached, resulting in 25 interviews; most participants were women (15 participants [60%]), reported primarily treating adult patients (15 participants [60%]), had more than 10 years of clinical experience (16 participants [64%]), and had submitted at least 2 single-patient EA requests to their institutional review boards during the relevant period (14 participants [56%]). Oncologists viewed EA as an important tool for securing what they determined to be the best treatment option for their patients based on their own expert assessment of available data. Interviewees reported that they would rather access interventions as commercially available products or through clinical trials; however, if the preferred option was not available through these means, they viewed pursuit of EA as part of their obligation to patients, while often recognizing the potential for inequities in the broader patient population beyond their institutions. Participating oncologists felt confident pursuing investigational drugs for treatment use, despite the absence of FDA marketing approval, and did not necessarily view EA as a last resort.

**Conclusions and Relevance:**

These findings indicate that oncologists practicing in large academic settings sought to treat patients with the interventions they deemed most likely to be beneficial, regardless of approval status. As such, they viewed EA as an unexceptional means to obtain promising products, although it remains unclear whether their confidence in evaluating investigational treatments was justified. Future research should examine whether oncologists outside large AMCs share this confidence, as differences may influence patient access to the EA treatment pathway.

## Key Points

**Question:**

How do oncologists at large academic medical centers with experience pursuing expanded access (EA) to investigational medicines perceive this regulatory pathway and their role in it?

**Findings:**

This qualitative study involved semistructured interviews with 25 adult and pediatric oncologists at 4 academic medical centers. Oncologists expressed confidence in determining whether an investigational treatment was the best option for their patients, based on their own experience and assessment of available data, independent of US Food and Drug Administration (FDA) approval status; when an investigational drug was determined to be the best option and trial enrollment was unavailable, oncologists indicated an obligation to pursue EA.

**Meaning:**

These findings suggest that academic oncologists valued evidence in recommending treatments but did not report feeling compelled to wait for marketing approval from the FDA, raising questions about access to EA in community settings where oncologists may be further removed from emerging evidence.

## Teaser

This qualitative study uses semistructured interviews to analyze the perspectives of academic oncologists on expanded access to investigational drugs and their role in offering patients these treatments.

## Introduction

The US Food and Drug Administration (FDA) expanded access (EA) pathway allows patients with serious conditions to access unapproved medical products for treatment use when certain requirements are met, including lack of satisfactory alternatives, potential benefits outweighing risks, and inability to access the intervention of interest through trial enrollment.^1^ Companies are not required to provide their products via EA, but if they do, the proposed use must also be reviewed by FDA and an institutional review board (IRB). Although the FDA rarely denies permission for single-patient EA requests,^2^ a second regulatory pathway, Right to Try (RTT), allows companies to provide investigational products outside trials without FDA oversight.^3^ To date, use of RTT appears infrequent.^4^

The rationale behind both pathways is similar: patients who are seriously ill may have insufficient time to wait for marketing approval of novel products and higher tolerance for risk.^5,6,7,8,9,10,11,12,13,14,15,16,17,18^ However, there are also concerns.^7,8,9,11,12,14,15,16,19,20,21,22,23,24,25,26,27,28,29,30,31^ Novel interventions may not work and can pose serious risks. Nontrial access may be inequitably distributed and might impede companies’ ability to gather safety and efficacy data. Additionally, the very idea of preapproval access may undercut the perceived need for marketing approval.

The FDA’s role as gatekeeper of investigational products has received substantial attention, while high-profile company refusals of EA requests have highlighted their involvement.^32,33,34,35,36^ Less is known about the role and perspectives of physicians regarding the use of unapproved medical products, although this is becoming an area of increasing study. Physicians serve as gatekeepers to unapproved products by suggesting use of specific interventions, navigating access pathways, and liaising with companies, IRBs, and regulators—or not. Oncologists are of particular interest: cancer care is a common indication for which EA is pursued, and the FDA’s Oncology Center of Excellence recently launched a program to assist oncology professionals with EA.^37,38,39,40^

Two recent studies of pediatric hematologists and oncologists found that comfort with the EA process varied and that many physicians—even at National Cancer Institute Cancer Centers—were unfamiliar with necessary regulatory steps.^41,42^ A survey of community oncologists found that nearly half reported having pursued EA, with success rates similar to FDA’s overall EA authorization rate, yet they struggled to distinguish between EA and RTT.^43^ Interview studies with clinicians outside the US demonstrate a mix of support for and concern about early access, alongside identification of practical barriers.^44,45^ Barriers identified in interviews with US clinicians include time and resource constraints, limited interest from pharmaceutical companies, lack of physician awareness, and lack of institutional support.^46^ Finally, a recent interview study of oncologists at Mayo Clinic sites examined both their perceptions of patient rationales for seeking investigational drugs and factors influencing oncologists’ decisions to request them.^47,48^

To build on this emerging literature and explore novel areas, especially regarding the relevance of FDA approval to oncologists’ treatment decisions, their perspectives on how EA fits into clinical care, and potential sources of inequities in access to EA, we interviewed a group of oncologists treating adult and pediatric patients at several large academic medical centers (AMCs) in the northeast United States.

## Methods

This qualitative study was deemed exempt from review by the University of Pennsylvania’s IRB and approved by NYU Langone Health’s IRB. Verbal consent was secured at the start of each interview. Study methods are reported following the Consolidated Criteria for Reporting Qualitative Research (COREQ) reporting guideline.

### Participant Recruitment

Using purposive sampling, we recruited adult and pediatric oncologists who had submitted at least 1 single-patient EA request to their IRB from January 1, 2014, through January 31, 2020. We secured lists of investigators meeting inclusion criteria from IRBs at the University of Pennsylvania (Philadelphia, Pennsylvania), Children’s Hospital of Philadelphia (Philadelphia, Pennsylvania), NYU Langone Health (New York, New York), and Dana-Farber Cancer Institute (Boston Massachusetts). Potential interviewees were invited to participate via email, including an information sheet and $50 gift card offer, with 1 to 2 reminders (University of Pennsylvania: 23 individuals; Children’s Hospital of Pennsylvania: 18 individuals; NYU Langone Health: 7 individuals; Dana Farber Cancer Institute: 52 individuals).

### Data Collection

A semistructured interview guide (eAppendix in the Supplement) was developed based on relevant literature and author experience as members (A.B.-H., L.K., and H.F.L.) of the NYU Grossman School of Medicine Compassionate Use and Preapproval Access Working Group.^6,7,8,9,11,12,13,14,15,16,17,20,21,22,25,26,27,28,29,30,35,37,40,41,42,44,45^ The guide was revised after pilot testing and encompassed the following domains: oncologist practice demographics and experience with EA; factors relevant to decisions to pursue EA and comfort with those decisions; perspectives on oncologists’ role in EA; EA-related experiences with companies, the FDA, IRBs, and AMCs; perspectives on the FDA’s role; and RTT. Interviews of approximately 60 minutes were conducted by a single interviewer (H.F.L.) both in-person and via videoconference from February 2020 to September 2021 until thematic saturation was reached.

### Data Analysis

Data were analyzed using thematic analysis.^49^ Using an essentialist-realist epistemological stance,^49^ the study team jointly developed a codebook based on the interview guide and adjusted to capture additional analytic elements across the data set. Deidentified transcripts were uploaded to Dedoose qualitative analysis software, and each was coded deductively by 2 of us (paired as T.S. and H.F.L., L.K. and H.F.L, or T.S. and A.B.H.). Discrepancies were resolved through discussion between coding pairs. If consensus was not reached, the senior author (H.F.L.) made a final determination. Codes were aggregated and grouped analytically to generate summary reports. Three of us (P.G., T.S., and H.F.L.) reviewed summary reports and identified themes. Data were analyzed from July 2021 to March 2022.

## Results

Of 100 invited oncologists, 53 did not respond, and 21 declined. We conducted 26 interviews and included 25 in the final analysis. Because only 2 interviews were completed at 1 institution, we initially excluded them both from analysis; however, the single interview conducted with a pediatric oncologist at that institution was ultimately included to address lower representation from pediatric practitioners in the overall sample of participating oncologists. Accordingly, adult oncologists were in practice at 3 sites, while pediatric oncologists were drawn from all 4. Most participants were women (15 participants [60%]), reported primarily treating adult patients (15 participants [60%]), and had more than 10 years of clinical experience (16 participants [64%]), with a range of 3 to 33 years. All participants indicated that they were frequently involved in clinical trials as either primary investigators or coinvestigators. Most had submitted 2 or more single-patient EA requests to their IRB during the relevant timeframe (14 participants [56%]), with a range of 1 to 7 requests. Participant demographics are further summarized in Table 1. We found several key themes, summarized with representative quotes in Table 2.

**Table 1.** Demographics of Participating Oncologists

Category	Oncologists, No. (%)
Clinical experience, y	
0-5	4 (16)
6-10	5 (20)
11-15	2 (8)
16-20	6 (24)
>20	8 (32)
Single-patient expanded access requests to IRB, No.^a^	
1	11 (44)
2	7 (28)
3	4 (16)
4	1 (4)
≥5	2 (8)
Type of oncology practice	
Adult	15 (60)
Pediatric	10 (40)
Gender	
Men	10 (40)
Women	15 (60)

Abbreviation: IRB, institutional review board.

^a^ Oncologists were invited to participate if they had submitted an expanded access request from January 1, 2014, to January 31, 2020.

**Table 2.** Summary of Key Themes and Representative Quotes

Theme	Summary	Representative quote^a^
EA: a means to an end	Oncologists at academic centers view EA instrumentally, as just another option for obtaining what they view as the best course of treatment for their patients.	“I want the best for my patients, and whatever, in this case, drug, is necessary to do that, I want to get. And as we’ve said, I click through options and [if] this rises to the top, you need to get it before it qualifies, if possible.” (A01)
		“The way that I think about expanded access, I don’t think about it as research. It’s an access mechanism. Is there a drug you think is going to help somebody? How do you get access to it? And if this is the only access mechanism, then I would consider it.” (A07)
		“It’s simple. If there is an agent that will help your patient, you are obligated to provide a mechanism to get that patient that agent.” (A14)
		“I think our role is to give the patient what we think is going to be the most effective and least toxic therapy and to get that therapy for them from whatever route is necessary. And so if that’s through a product that’s not yet been approved, then that’s still my role.” (P10)
		“And so, for me, expanded access is just another tool to convey to families that we want to do everything we can to care for the patient and to care for them. And it’s just another way to care for them. And sometimes it’s the right way, and sometimes it’s not going to be helpful.” (P07)
Making treatment decisions: the centrality of data	When considering treatment options, academic oncologists rely heavily on data. In the face of uncertainty, they prefer recommending that patients enroll in available trials.	“Physicians have an obligation to carefully consider whether giving an unapproved drug is the right thing. I know that people get compassionate use for patients where there’s a clinical trial available because the clinical trial randomizes the drug vs placebo. I have never offered a patient compassionate use in that setting because I feel like you have to answer the question. If we knew the right answer, everybody would have access to the drug.” (A02)
	EA may not be a last resort, if data suggest that an investigational product is the best option for a given patient.	“I’ll say that it’s not necessarily only in people who have exhausted their standard options. If there’s somebody with a target where I think the targeted therapy is better than standard therapy, I would consider a single-patient IND before their standards are exhausted.” (A15)
	Academic oncologists express strong confidence in their ability to parse data to decide on the most beneficial therapy, independent of FDA approval status.	“I really am pretty much of a stickler that FDA approval is something that’s given to the drug companies to market a drug. It has nothing to do with whether it’s given to a physician to prescribe the drug, right? So we use a compendium of public literature [and] phase 2 and 3 trials to decide whether to treat somebody with something as a marker of efficacy. But, I always tell my trainees that the FDA approval is the approval to market for an indication, it’s not approval for physician use… I’m not given approval to use something. I get that from my MD.” (A14)
	Academic oncologists still value FDA approval, even as they pursue EA.	“The other reasons why we may pursue expanded access is primarily the strength of the emerging data that’s out there, conference abstract presentations, which is really the main source of data for agents that are not approved yet, or if the paper’s been published.” (A08)
		“Since at [institution] we’re obviously involved in some of the early trials with the key drugs, we tend to know about these, many patients who we think would benefit from these drugs based on their mutational profile or their disease. If they’re between a phase 1 trial and a phase 3 approval trial, then the only way to get the drug is through compassionate use, and that’s when I’ll bring it up.” (A12)
		“If I think that something sounds like an interesting therapeutic option for a patient and I am aware the data suggests that there’s a reasonable risk-benefit and I can get it, which is the hard part… So how long do they have to wait is driven entirely by data. New mechanism of action. Some evidence in kids that it works. … I think it’s more data-driven than time or where it is in the clinical process.” (P02)
		“I think the approval process is important to define safety, and I think that it’s important to define efficacy, and I think that we should not be using unapproved drugs widely without understanding what the risks and benefits are of those drugs.” (A07)
		“I think one of the more obvious logistical reasons [that FDA approval is needed] is that there are just not enough resources in the world to go through this process for each patient every time that you’re using a drug. And so the way to streamline that is to get a blanket approval for the drug, so that we can use it in all patients.” (P04)
Deciding to pursue EA: weighing risks, benefits, and burdens	When considering EA, oncologists recognize that the benefits of investigational medical products are uncertain, but they will not pursue EA without some reason to expect benefit and will refrain when the risks of harm are substantial.	“We’ve seen again and again drugs that look good in phase 2 trials, [but] in phase 3 trials either the efficacy didn’t look so good or there were some terrible unexpected toxicities… It is the nature of most to overestimate benefits and underestimate harms from investigational compounds.” (A02)
	Oncologists treating pediatric patients were more likely to consider pursuing EA even with low likelihood of benefit, in part to support parental desire to “leave no stone unturned.”	“I would never go through this process for the sake of just having something to give [the patient]. It really has to be pretty strong evidence that it’s likely to work, or at least has, yeah, or at least has a good chance to work.” (A12)
		“And when the risk of doing actual harm is high, it’s not something that I can recommend to an individual patient.” (P08)
		“In pediatrics, I think a lot about parents who are going to survive their children, and they need to live the rest of their lives and be comfortable with the decisions they made around their child’s death. And so I tend to be a little more liberal with what I offer and allow at the end of a child’s life, to make sure that parents don’t have regrets or guilt, or have as little as possible.” (P09)
EA within the physician-patient relationship	Oncologists report that discussions about EA are typically physician-initiated.	“I’ve had it happen extremely rarely that patients are asking me about drugs if I’m not bringing it up. I’m the initiator here, so I’m not even going to talk about compassionate use unless I think it’s valuable.” (A02)
	In patient discussions about EA, strong disagreements rarely occur and are usually surmountable with attention to clear explanation and demonstrated support.	“Those discussions are often very hard, but often, with a lot of discussion, families, most families, will understand that causing side effects could end their child’s life sooner than would otherwise have happened.” (P08)
	When disagreements persist, patients may seek EA through a different physician.	“I think that patients and their families are afraid of dying, and they’re afraid of being abandoned or left alone in that process. And so, I can’t control whether they live or die, but I have more control over how I position myself and people with whom I work in terms of supporting families so that they are less likely to feel that they’re going to be abandoned or that they’ll be alone in this process.” (P07)
		“I do not feel that I ever have any obligation to do compassionate use even if the patient wants it, and they can always find a different physician if I do not feel that it’s in their best interest to do so, that there’s other doctors that might do it for them, but I don’t have to.” (A02)
		“I have had families say that they would like to switch providers to see if they could find a provider who would do something compassionately or on expanded access.” (P08)
Inequities in access	Oncologists acknowledge structural inequities in accessing the EA pathway.	“There are also equity issues just in terms of where people are seen, because the patients I’ve applied for have been because I have known of some drug or whatever, because I’m an investigator on various trials. So, because they’re seen in an academic center, their doctor has knowledge of some things that other people may not.” (A02)
	Oncologists generally report the provision of unbiased access to EA for their own patients.	“Because a patient at some level does have to advocate a little bit for him or herself and that is just going to be more possible, greater likelihood of that with higher education, higher socioeconomic status, a better network, a better connection, better connections to everything. I really don’t know what to do about that.” (A03)
		“I would hopefully think I would to be as likely to bring it up regardless of the gender, ethnic, racial, socioeconomic issues with the patient. Obviously, I’m a human being, and that’s probably not completely true.” (A12)
		“I don’t think there would be any issue of fairness because we as the provider would be the one who would have to do the process and there’s no financial commitment from the patient whatsoever. There might be a difference in which patients and families are pushing for it based on their education level and their awareness, but ultimately, it’s our responsibility to say yes or no and pursue it.” (A06)
		“If a family comes and asked me about a drug, they have as much chance of getting that drug as anyone else, regardless of the resources on their part.” (P01)
		“I think in a micro-environment, when I think about my own little practice, expanded access... everybody’s treated the same. The protocols you write, they’re just like treating people with anything else.” (A07)

Abbreviations: A, adult oncologist; EA, expanded access; FDA, US Food and Drug Administration; IND, investigational new drug application; P, pediatric oncologist.

^a^ For anonymity, respondents are identified by number and whether they were a pediatric or adult oncologist, without reference to their specific institution.

### Expanded Access: a Means to an End

Although EA is an exceptional regulatory pathway permitting access to unapproved products, oncologists in our sample viewed it as squarely within the range of available and reasonable treatment options. They reported focusing first on identifying the therapeutic agent most likely to benefit a patient and only then considering how to secure access. Decisions to pursue EA were considered merely incidental, a means to an end necessitated by the fact that the product deemed the best treatment option had not yet been approved by the FDA and was not available to the patient via a trial. In this regard, oncologists largely viewed EA simply as a tool in their toolkit, different in process but not intention from other options for securing patient treatments.

### Making Treatment Decisions: the Centrality of Data

Participating oncologists often indicated a preference for using approved drugs or enrolling patients in trials before pursuing EA. Underlying these preferences was a strong reliance on data to guide oncologists’ clinical recommendations, with approved products recognized as having the strongest evidentiary support. In the face of uncertainty, oncologists indicated that trial participation was the best option for the benefit of both current and future patients, producing critical data about safety and efficacy. Yet several oncologists indicated that EA was not always perceived as a last resort. They explained that EA may be appropriate even if approved drugs or trials of other investigational products are available, so long as data indicate that the intervention sought via EA would be the patient’s best option. Oncologists reported using all available data to inform EA recommendations, including evidence from early-stage trials, unpublished data provided by sponsors or shared at scientific meetings, evidence of success from a patient’s previous trial participation, or a strong scientific rationale for a product’s efficacy.

Overall, oncologists in our sample expressed confidence in their abilities to analyze data in evaluating investigational treatment options, making what they perceived to be informed recommendations even before products were evaluated by the FDA. Relatedly, a few oncologists reported that they would consider using RTT (bypassing the FDA) if that pathway provided a simpler means of access, although they largely expressed opposition to the idea of patients having a genuine right to demand specific interventions. Oncologists’ reliance on their own parsing of data did not suggest that they rejected the importance of requiring FDA marketing approval. Rather, they appeared to value this requirement instrumentally because it requires companies to generate evidence that in turn provides oncologists the greatest confidence.

### Deciding to Pursue Expanded Access: Weighing Risks, Benefits, and Burdens

As in any other treatment decision, oncologists in our sample indicated that EA involves weighing risks and benefits. However, the typical uncertainty surrounding investigational products can make this analysis harder. Pediatric oncologists noted this challenge acutely, given that there are usually less data available in children.

Oncologists reported pursuing EA only when there is some evidence the investigational product could offer benefit. While patients may initially express a desire to “leave no stone unturned,” most adult oncologists did not find this a compelling reason to pursue EA. Pediatric oncologists were more likely to be receptive to requests to try long-shot efforts, in part to support parents.

In addition to weighing risks and benefits, oncologists in our sample also considered different types of burden. Nearly all reported considering the additional professional effort that accompanies EA, while noting it would not prevent them from using this pathway if needed. In line with the fact that interviews were conducted exclusively at large AMCs, participants commonly noted that they were able to rely heavily on institutional staff assistance, typically from clinical research personnel, when pursuing EA requests, especially to help with paperwork and regulatory requirements. A few oncologists, especially in pediatrics, reported considering additional burdens on patients and families, such as extra visits; however, none reported this as a major factor in deciding whether to pursue EA. The Figure summarizes the factors described as playing a role in interviewees’ EA decisions.

**Figure.**
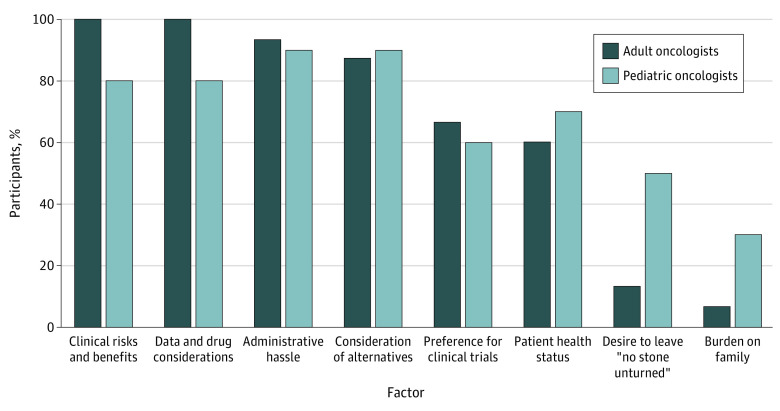
Oncologist-Reported Factors Influencing Decisions to Offer Expanded Access Statistical significance was not calculated for differences between adult and pediatric oncologists in this small qualitative sample; this descriptive comparison is presented only for the purpose of generating hypotheses for future research.

### Expanded Access Within the Physician-Patient Relationship

Oncologists reported that they were almost always the party who initially raised the idea of EA and they tended to be cautious, bringing this up only when convinced there was a valuable and realistic intervention to offer. Some oncologists contacted companies to learn their EA policies before discussing the option with patients. In the rare instances that EA conversations were patient-initiated, oncologists typically described those patients as savvy and well-resourced.

Oncologists indicated that because they are well-practiced in difficult conversations, discussions about EA were not uniquely challenging, raising familiar issues about false hope and uncertain benefit. Similarly, when asked about the physician’s role in EA, oncologists described roles that are not unique to the EA context but can be exacerbated in that setting (Box).

Box. **Representative Quotes on Academic Oncologists’ Perspectives on Their Role in EA^a^**

**Nothing Unique**

“The physician’s role in expanded access is the same as the physician’s role in all other aspects of patient care. You have to be an honest broker for your patient and do what you can based on your knowledge and the knowledge of your colleagues who you may ask how best to treat the patient.” (A12)

**Advocate**

“I think there’s an advocacy component, where if there’s sufficient information or data to support the use of something in a certain population, it at least should be thought of.” (A13)

**Source of Information and Advice**

“I think 1 role is to evaluate what makes sense to go for, because I do think that it’s difficult for patients to really necessarily sift through the data.” (A11)
“I think [the role is] deciding… whether it’s appropriate to offer something, and then explaining to the patient what this program [EA] is, and what the drug is, and why we think it’s appropriate to offer it.” (P09)

**Expectation Manager**

“At least in my world of cancer, you just have to be careful about not over-promising. So I think you want to avoid being too enthusiastic because that’s actually a bit of a cop-out. If you sort of oversell the expanded access program to the patient as a way of avoiding your own responsibility for thinking about alternatives, like clinical trials, hospice, et cetera, you raise hope, you raise expectations.” (A03)

**Gatekeeper**

“I don’t think it’s our job to go after… every drug that’s out there that could possibly be effective. Kind of the long-shot, kind of crazy therapies that… I don’t think it’s our job to explore every single possible drug for a patient.” (P10)

Abbreviations: A, adult oncologist; EA, expanded access; P, pediatric oncologist.

^a^
For anonymity, respondents are identified by number and whether they were a pediatric or adult oncologist, without reference to their specific institution.

Oncologists reported that disagreements with patients seeking investigational products against the oncologist’s recommendation occurred infrequently and could typically be resolved without relational harm by spending time listening to patients, understanding their circumstances, emphasizing they would not be abandoned, and being direct about why investigational options would not likely help. Several oncologists reported that patients often lost interest in pursuing EA once they understood the low likelihood of benefit.

### Inequities in Access

Most oncologists acknowledged barriers to EA that could lead to inequities. These included access to a physician with necessary knowledge to identify appropriate investigational treatments, often derived from running trials and being at the leading edge of cancer treatment; receiving care at institutions with resources to support EA; differential knowledge and ability to self-advocate across patients; and challenges associated with extra visits and adherence to EA logistical requirements. Oncologists noted that costs were not likely a source of inequity in EA, for several reasons: patients pursuing EA are usually at an advanced stage of illness with higher rates of clinical engagement in any event; insurers typically cover costs of ancillary care beyond the EA product; and despite the legal permissibility of companies charging direct costs for EA products, none of the oncologists in our sample had experienced this.^50^ Pediatric oncologists sometimes noted unique structural supports, including insurance coverage and institutional resources, for families of a child undergoing cancer care. Despite recognizing structural inequities, most oncologists perceived themselves as pursuing EA without individual bias.

## Discussion

In this qualitative study interviewing oncologists at large AMCs who had prior experience with EA, we found that they often viewed the EA pathway like any other treatment option, focusing primarily on identifying the best treatment recommendation and relying on their own assessment of available data, with only secondary attention to approval status and the pathway through which the product may be accessed. Although treatments with strong evidence and trial participation are both typically preferred to providing investigational medical products through EA, oncologists in our sample were comfortable with decision-making in the face of uncertainty and would pursue EA when they deemed the risk-benefit balance more favorable than other options. Like other areas of medicine, oncologists recognized that EA may be most accessible to the privileged.

Our findings align with results of previous interview studies, while offering novel insights. Similar to the findings of a 2021 study by Stout et al^48^ among oncologists at the Mayo Clinic, we found that oncologists in our sample were typically the initiators of EA discussions with patients; were usually able to manage patient expectations about investigational drugs; considered scientific rationale, risk:benefit ratio, patient functional status, and alternative treatments as key factors in making EA decisions; and perceived a duty to pursue EA when it would benefit patients, while rejecting the notion that patients have a “right to try” any intervention they desire.^35^ In a study of Dutch physicians, Bunnik and Aarts^20^ similarly documented the perspective that pursuit of investigational treatments is a core physician obligation for patients without other options when there is adequate evidence to determine likely benefits and risks, but that access to investigational products should not be provided just to give hope.

Whereas Bunnik and Aarts^20^ found that physicians only rarely pursued nontrial preapproval access without exhausting other options, several oncologists in our sample suggested that a product available only through EA could be the best option even if others had not yet been tried. Although it may be true in any given case that standard of care options are unsatisfactory, fulfilling an important eligibility criterion for EA,^51^ this perspective could inhibit the ability to gather data about other investigational treatments through trials for which the patient may be eligible. Further empirical and normative probing of this finding is warranted: how do oncologists understand regulatory and ethical requirements for EA eligibility, might they sometimes perceive them to conflict with their clinical obligations to patients, and to what extent should EA be a true last resort? Given that regulatory criteria regarding the impact of EA on trials focus exclusively on the requested product,^51,52^ the FDA should also consider offering guidance regarding pursuit of EA for a particular investigational product vs participating in a trial of another.

The most important new finding from our interviews is how confident participating oncologists were in assessing treatments that were not FDA-approved. This was not an indication that oncologists viewed evidence as unimportant; to the contrary, data considerations were among the most important factors in their EA decisions. However, academic oncologists described having access to various data sources about investigational drugs and did not feel compelled to wait for or rely on FDA’s assessment through marketing approval.

This finding has at least 2 implications. First, it leads to additional questions. Do academic oncologists truly have access to adequate data to make reasonable recommendations about unapproved products? How accurate are their projections about benefit and risk in these contexts? The study by Stout et al^48^ found that most oncologists in their sample reported moderate, little, or no benefit for patients administered EA drugs, although some saw dramatic responses, while a study by Chapman et al^41^ found that most surveyed pediatric hematologists and oncologists reported psychological benefits for families but fewer reported psychological and clinical benefits for patients. Do these findings reflect poor projection of benefit given limits on available data, acceptance of low possibility of benefit given lack of alternatives, or both? Although these are open questions, oncologists in our sample recognized the uncertainty of treatment with investigational products and conveyed that to patients. They also valued trials as the best way to determine a treatment’s efficacy.

Second, reported confidence in evaluating investigational options raises additional concern about the role of privilege in EA. Because oncologists at AMCs may be running trials, practicing in centers with substantial research portfolios, reviewing grant applications and manuscripts, presenting at conferences, and liaising with companies, they likely have more extensive access to the limited data that exist about investigational drugs than physicians practicing in other settings. If data are critical to EA decisions and some oncologists are not deeply engaged with those data, then some patients’ access to investigational drugs will likely be negatively impacted, particularly most patients with cancer who receive care in community settings rather than at large, urban academic centers.^53^ Although the FDA has undertaken efforts to make EA more accessible, they do not address gaps in awareness of investigational options or ability to assess available data. Additionally, the fact that oncologists in our sample reported considerable administrative hassle despite extensive staff support suggests another source of likely EA disparities for patients receiving care in community settings with fewer resources.

Finally, we note that there may be advantages and disadvantages to the perception that EA is “just another tool” for giving patients the best treatment. The upside of this view is that oncologists who hold it are willing to make use of the EA pathway, which offers some prospect of benefit in the right circumstances, although it is more burdensome than routine clinical care. The downside is that it may mask the fact that EA is supposed to be exceptional: it involves treating a patient without the research goals that justify the risks of offering unproven treatments in trials and without the data that justify treating patients with approved interventions. Oncologists should be willing to use EA, but only with appropriate caution. Notably, oncologists participating in our study did acknowledge the weightiness of this regulatory pathway.

### Limitations

This study has some limitations. Our sample was limited to oncologists at a small group of large AMCs; oncologists in less research-intensive settings and with fewer resources may have different perspectives. Our sample likely also differs from oncologists who have not previously used EA. Although interviewees were asked to focus on single-patient EA, they also sometimes discussed EA for larger cohorts and off-label prescribing, possibly indicating confusion regarding relevant pathways, also seen in prior literature.^41,42,43^ Where confusion was apparent during interviews, clarification was sought.

## Conclusions

This qualitative study found that academic oncologists with EA experience report taking their gatekeeping role in this pathway seriously. Although they placed greater emphasis on determining the best treatment option for patients than on the pathway through which it may be available, they would pursue EA only when their expert assessment of available data indicated reason to believe the benefits of treatment with an investigational product outweighed risks. Given implications for both patient access to and benefit from EA, future research should examine whether this self-confidence in assessing investigational products is justified, as well as how it compares to the views of those without EA experience and practicing outside academic settings.

## Notes

Supplement. **eAppendix.** Interview Guide
