# Supplementary material for: Perspectives of Academic Oncologists About Offering Expanded Access to Investigational Drugs
Source: JAMA Netw Open. 2022 Nov 1;5(11):e2239766. doi: 10.1001/jamanetworkopen.2022.39766 (PMC9627412; doi:10.1001/jamanetworkopen.2022.39766)
Supplement: Supplement. — eAppendix. Interview Guide [file jamanetwopen-e2239766-s001.pdf]

Supplemental Online Content

Gould P, Salam T, Kimberly L, Bateman-House A, Fernandez Lynch H. Perspectives of academic oncologists about offering expanded access to investigational drugs. *JAMA Netw Open*. 2022;5(11):e2239766. doi:10.1001/jamanetworkopen.2022.39766

eAppendix. Interview Guide

This supplemental material has been provided by the authors to give readers additional information about their work.

© 2022 Gould P et al. *JAMA Network Open.*

eAppendix. Interview Guide

Introduction

- I'm [NAME] and I'm a [POSITION] at [INSTITUTION]. [INTRODUCE ANYONE ELSE JOINING INTERVIEW.] Thank you for agreeing to this interview and for taking time out of your day to speak with me.

- So that we can create an accurate transcript of what we discuss here, I'd like to record our conversation. Do I have your permission to do so?
  - *[Wait for confirmation. Begin recording. State interviewer name, date, participant ID.]*

- Thank you. The purpose of this study is to learn from oncologists who have been involved in facilitating access to investigational drugs outside clinical trials about how they perceive their role and the roles of other stakeholders, including patients, pharmaceutical/biotech companies, the FDA, and IRBs. Our hope is that this information will help institutional and regulatory policymakers think about how to best support physicians considering this approach.

  - When we discuss Expanded Access in this interview, we are referring to the pathway sometimes referred to as compassionate use, in which a company agrees, with permission from the FDA, to provide a patient with pre-approval access to an investigational drug outside a clinical trial.

  - We are interested specifically in Expanded Access requests for single patients, as opposed to intermediate-sized populations or widespread treatment use.

  - We will also ask a few questions about the pathway known as Right to Try, in which permission from the FDA is not required.

  - Our intention is to focus on non-trial use of products that are not yet approved by FDA for any indication, but we understand that sometimes the Expanded Access pathway may be used for off-label products as well, especially in pediatrics. If you have experience in that regard, please let us know so we can make sure to be clear about what type of Expanded Access requests you mean to refer to.

  - Is all of that clear? Do you have any questions? *[Wait for response.]*

- Before we begin, I want to remind you that your participation is voluntary, and we will take steps to protect your privacy. You can skip any question, decide to end your participation at any time, or ask at the end of the interview that we not use your data for our project. We will create a transcript of the interview, remove any identifying information, and then destroy the audio-recording when the study is over. When we publish our results, we will not attribute any quotes to you or your institution.

- Would you like to proceed? *[Wait for confirmation.]*

Let's start with some information about you, your clinical practice, and your involvement with research.

1. How many years of experience do you have as a practicing oncologist post-fellowship?

2. Do you see adults or children in your clinical practice, or both?

3. If you think about the 6 years just before the pandemic, so from about 2014 to the start of 2020, would you estimate that you have been involved in clinical trials as a PI or investigator frequently, occasionally, or never?

4. Approximately what percentage of your patients would you estimate are currently enrolled in a clinical trial? And about what percentage would you estimate has ever participated in a clinical trial during the course of their cancer?

5. Approximately what percentage of your patients would you describe as having exhausted standard treatment options? And how would you define exhaustion of standard options in this context?

Let's talk now about your experience with Expanded Access.

6. Again thinking about the 6 years just before the pandemic, 2014 to the start of 2020, for about how many of your patients would you estimate that you have discussed Expanded Access with the patient and/or their family?

7. How does this typically come up?
   a. How often are you raising the issue?
   b. How often are patients and their families raising it?
   c. Does it ever come up in a different way, and if so, how?

8. Have you seen any change in the frequency of these discussions over time?
   a. If so, what do you think explains that? *[Probes, if relevant: Why are you bringing it up more often? Why do you think your patients are bringing it up more often?]*

9. How many times in the 6 years just before the pandemic would you estimate that you have engaged in discussions with a pharmaceutical/biotech company about Expanded Access for one of your patients?

10. How many times in the 6 years just before the pandemic would you estimate that you have submitted an application to the FDA for Expanded Access for one of your patients?

Now let's dig a bit deeper into how you think about Expanded Access.

© 2022 Gould P et al. *JAMA Network Open.*

11. Would you describe your understanding of the Expanded Access process as strong, moderate, limited, or very minimal?

12. Do you recall when and how you first learned about the Expanded Access pathway? If so, can you tell me about it?

13. What factors would make you likely to consider Expanded Access as an option for one of your patients. Why? *[If needed, prompt with examples like availability of alternatives, clinical trial eligibility, level of evidence, concern about harm, etc.]*

14. And what factors would make you unlikely to pursue Expanded Access for one of your patients?

    a. In these cases, what might you recommend? For example, waiting for an investigational product to be approved by FDA, pursuing palliation rather than curative treatment, or something else altogether.

15. When considering Expanded Access for one of your patients:

    a. Do you think about the interests of your individual patient compared to how Expanded Access might affect future patients as a group? Please explain. *[Further clarification if needed: For example, do you think it is important to consider how Expanded Access might influence FDA approval?]*

    b. Do you think about which social factors might make some patients more or less likely to be able to secure Expanded Access, as a matter of fairness? Please explain.

    c. Do you think about the financial implications of Expanded Access for patients, for example the need to be able to pay any costs of the investigational product or for additional clinical visits, and things like that? Please explain. *[If the response is that their patients never pay, ask: Would it change your mind if you knew that your patient would have to pay out of pocket to access the investigational product?]*

    d. Do you think about the possibility of encouraging false hope? Please explain.

    e. Is there anything else that you might have on your mind when thinking about Expanded Access as a possible option for one of your patients?

16. Have you ever had a patient who wanted to seek Expanded Access when you believed it wasn't appropriate? If yes, how did you handle it?

© 2022 Gould P et al. *JAMA Network Open.*

    a. Do you think about preserving a good doctor-patient relationship? Please explain.

17. In general, do you feel comfortable making decisions about Expanded Access? Why or why not?

18. How would you describe the physician's role with regard to Expanded Access? [*If there is confusion/hesitation, give examples, e.g., do you view yourself as having a role in serving as a source of information to patients about unapproved products, making clinical judgments about whether Expanded Access is appropriate for particular patients, or serving as an advocate to help patients secure Expanded Access?]*

19. Is there anything you think the physician's role should <u>not</u> entail when it comes to Expanded Access?

20. *[For interviewees who treat both adults and children]* Would any of your perspectives on Expanded Access change if you were thinking of an adult patient versus a pediatric patient or vice versa?

I'd like to move on to discuss your experience and thinking about <u>pharmaceutical/biotech companies and Expanded Access</u>.

21. When you have sought Expanded Access from a company, about how long did you (or your staff) spend on any paperwork required by that company? [*Clarify that this is only time spent on materials required by the company, not paperwork submitted to FDA, the IRB, etc.]*

22. Start to finish, from the time you first contacted the company till it made its decision, about how long did the process take?

23. About how often (if ever) have you had a company <u>decline</u> to provide Expanded Access for one of your patients?
    a. Did it provide a reason? If so, what was it?
    b. How did you respond?

24. What reasons would you find <u>acceptable</u> for a company to decline providing Expanded Access?

25. What reasons would you find <u>unacceptable</u>?

Now let's discuss how you think about the <u>FDA and Expanded Access</u>.

26. What value, if any, do you see in the FDA's involvement in reviewing Expanded Access requests? *[e.g., beyond what the interviewee already knew, what the company added, etc.]*

© 2022 Gould P et al. *JAMA Network Open.*

27. What frustrations or concerns, if any, do you have about the FDA's role in Expanded Access?
    a. Do you think that the FDA imposes any review criteria or requirements for Expanded Access that are unnecessary? If so, what are they?

28. Are you aware of FDA's pilot program, Project Facilitate?
    a. *[If yes, what do you think about it?]*
    b. *[If no, briefly explain that it is a concierge program for oncologists to seek assistance with the process of Expanded Access from a designated point of contact at FDA, who helps with things like applications and liaising with sponsors. Then ask: What do you think about that?]*

29. When you have submitted an application for Expanded Access to the FDA, about how long did you (or your staff) spend on the FDA paperwork? *[Clarify that this is only time spent on FDA materials, not company or IRB paperwork.]*

30. And about how long did it take for the FDA to make its decision?

31. What kinds of questions, comments, or changes did the FDA have, if any?

32. In general, how do you think the FDA should balance the interests of individual patients seeking Expanded Access with its public health mission to protect all patients through pre-approval requirements?

33. How would you respond to the following query, perhaps from a patient ineligible for Expanded Access: "If we allow some patients to access unapproved drugs outside of clinical trials, why should any patients have to wait for FDA approval?"

We're almost done. Now, I'd like to ask you about your <u>institutional environment and Expanded Access</u>.

34. What kind of resources, if any, are available at your institution to assist with Expanded Access requests? Who provides them?

35. Is there any process, formal or informal, for engaging with your physician peers about Expanded Access requests, such as a review committee or encouragement to seek a second opinion?

36. Have you encountered any barriers to Expanded Access at your institution? What are they?

© 2022 Gould P et al. *JAMA Network Open.*

37. When you have submitted an application for Expanded Access to the IRB, about how long did you (or your staff) spend on the IRB paperwork? *[Clarify that this is only time spent on IRB materials, not company or FDA paperwork.]*

38. About how long did it take for the IRB to make its decision?

39. What value, if any, do you see in having IRBs approve Expanded Access requests? *[e.g., in addition to the physician's own review, company input, FDA authorization, etc.]*

40. What frustrations or concerns do you have, if any, about the IRB's role in Expanded Access?

Before we wrap up, I'd like to ask you a few questions about the <u>pathway called Right to Try</u>.

41. Are you familiar with this pathway and how it differs from Expanded Access?
    a. If yes, can you briefly describe your understanding of the difference?
    b. [*Correct any stated misperceptions at the end of the interview.]*

42. *[Ask even if unfamiliar, so long as they have at least heard of RTT]* Have you ever discussed Right to Try with your patients? *If yes:*
    a. How frequently?
    b. How did it come up?
    c. How did the discussion differ, if at all, from conversations you've had with patients about Expanded Access?

[Penn and CHOP interviews included the following additional questions:]

Would you view your responsibilities as a physician any differently regarding Expanded Access versus Right to Try?

What is your general impression of Right to Try (e.g., is it helpful, harmful, etc.)?

General

43. How important do you think it is to collect safety data about patients using investigational products outside a clinical trial? What about efficacy data?

44. Do you think there are any barriers to collecting data about these patients?

45. When you've provided patients with access to an investigational drug outside a clinical trial, what have you been able to bill for?

46. Has your thinking about Expanded Access changed at all in the midst of COVID-19? If so, how? Why?

© 2022 Gould P et al. *JAMA Network Open.*

47. Have you noticed any change in the nature or frequency of your discussions about Expanded Access in the midst of COVID-19? If so, can you describe how these conversations have changed and with whom you've been having them (colleagues, patients, others)?

48. Is there anything else that you'd like to discuss that I haven't asked about yet?

Thank you for agreeing to participate in this interview. Your involvement will help us better understand how oncologists think about pre-approval access to investigational drugs outside clinical trials. We plan to publish the results of the study and will share publications as they are issued.
[Correct any stated misperceptions about EA or RTT after the interview closes.]

© 2022 Gould P et al. *JAMA Network Open.*
